# Group 2 innate lymphoid cells and their surrounding environment

**DOI:** 10.1186/s41232-023-00272-8

**Published:** 2023-03-20

**Authors:** Maiko Naito, Atsushi Kumanogoh

**Affiliations:** 1 Department of Respiratory Medicine and Clinical Immunology, Graduate School of Medicine, Osaka University, Suita, Osaka Japan; 2 Department of Immunopathology, World Premier International Research Center Initiative(WPI), Immunology Frontier Research Center (IFReC), Osaka University, Suita, Osaka Japan; 3 Integrated Frontier Research for Medical Science Division, Institute for Open and Transdisciplinary Research Initiatives (OTRI), Osaka University, Suita, Osaka Japan; 4 Center for Infectious Diseases for Education and Research (CiDER), Osaka University, Suita, Osaka Japan; 5 Japan Agency for Medical Research and Development–Core Research for Evolutional Science and Technology (AMED–CREST), Osaka University, Suita, Osaka Japan; 6 Center for Advanced Modalities and DDS (CAMaD), Osaka University, Suita, Osaka Japan

**Keywords:** Adventitial stromal cell (ASC), Group 2 innate lymphoid cell (ILC2), Mesenchymal cells, Microenvironment

## Abstract

Since the discovery of group 2 innate lymphoid cells (ILC2s) in 2010, subsequent studies have revealed their developmental pathways, mechanisms of activation and regulation, and immunological roles in tissue homeostasis and tissue-specific diseases in various organs. Although ILC2s are known to express tissue-specific features depending on where they reside, how the surrounding environment affects the functions of ILC2s remains to be fully elucidated. Recent histologic analyses revealed that ILC2s resides in specific perivascular regions in peripheral tissues with their function being controlled by the surrounding cells via cytokines, lipid mediators, neurotransmitters, and cell–cell interactions through surface molecules. This review summarizes the interactions between ILC2s and surrounding cells, including epithelial cells, neurons, immune cells, and mesenchymal cells, with the objective of promoting the development of novel diagnostic and therapeutic methods for ILC2-related diseases.

## Background

**Fig. 1:**
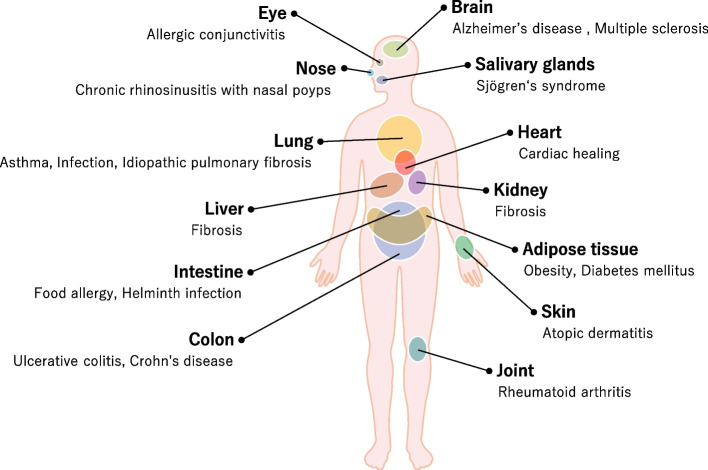
ILC2s and its related diseases. Studies with human and mouse revealed that ILC2s reside in various tissues exhibiting tissue-specific features and play a pivotal role in maintaining the homeostasis of tissues they reside

Group 2 innate lymphoid cells (ILC2s) are a subset of innate lymphoid cells that express transcription factor GATA-binding protein 3 (GATA3) and lack specific lineage markers [1]. Unlike adaptive lymphocytes, ILC2s do not possess antigen-recognition receptors such as the T cell antigen receptor or B-cell antigen receptor. Instead of antigen-specific responses, ILC2s respond to environmental factors including cytokines, neuropeptides, lipid mediators, hormones, and nutrients. In response to these inputs, ILC2s produce various cytokines, such as IL-4, IL-5, IL-6, IL-9, IL-10, IL-13, GM-CSF, and amphiregulin. By producing these cytokines, ILC2s are associated with the pathogenesis of various diseases, including parasitic infections, allergic diseases, autoimmune diseases, metabolic diseases, and malignant diseases (Fig. 1) [2–10]. ILC2s were first discovered in murine adipose tissue [2] but are now known to be present in various tissues in both humans and mice, for example, lung, intestine, brain, heart, liver, skin, and peripheral blood [11–17]. ILC2s are generally tissue-resident cells that possess tissue-specific functions. Progress in the past several years has clarified the factors that regulate the activation, suppression, expansion, development, and migration of ILC2s. However, how the surrounding environment affects tissue-resident ILC2s is not fully understood. This review summarizes recent advances in the understanding of mechanisms regulating ILC2s by surrounding cells, which would contribute to the development of basic studies on ILC2s and the investigation of new clinical targets for ILC2-associated disorders.

### Development and migration of ILC2s

ILC precursors and ILC2s first arise during fetal development, both in humans and mice. They are seeded in peripheral tissues and differentiate in situ to acquire tissue-specific profiles [18–20]. Shortly after birth, fetal ILC2s are followed by neonatal origin ILC2s, which comprise the majority of adult peripheral ILC2 pools accompanied by the acquisition of effector repertoires and tissue-specific signatures [21, 22].

In adult parabiotic mice, ILC2s were shown to be tissue-resident cells. They are maintained and expanded locally both during homeostatic conditions and acute helminth infection. However, during the chronic phase of infection, a fraction of ILC2s from hematogenous sources are recruited to replenish the pool of resident ILC2s, leading to the clearance of the infection and tissue healing [23]. In addition to helminth infection, IL-25 induces inflammatory ILC2s in the small intestines to proliferate and traffic to the lymphatics and blood circulation. They reach the peripheral tissues, including the lungs, in a sphingosine-1-phosphate-dependent manner [24]. Ricardo-Gonzalez et al. showed that the activation of local ILC2s by tissue-specific alarmins induces ILC2 proliferation, lymph node migration, and blood dissemination, which causes local innate responses to transition into systemic type 2 responses [25]. As mentioned above, ILC2s are basically tissue-resident cells; however, in certain conditions, they migrate from tissue to tissue and they turn local inflammation into systemic inflammation.

Recent studies have shown that tissue niches play a crucial role in the acquisition of tissue-specific properties by tissue-resident or migratory ILC2s. Using a single-cell atlas of lung ILC2s, Zeith et al. showed that tissue-resident *Il18r1*^+^ ILC2 precursors contribute to the maintenance and renewal of tissue ILC2s via in situ differentiation, suggesting that local niches, rather than origin or developmental period, might dominantly imprint ILC phenotypes in adult tissues [26]. For instance, neuropilin-1 (Nrp1) is a tissue-specific marker of lung ILC2s that is induced postnatally and sustained by lung-derived transforming growth factor beta-1 (TGFβ1). TGFβ1–Nrp1 signaling enhances ILC2 function and exacerbates bleomycin-induced lung fibrosis through upregulation of IL-33 receptor ST2 expression [27]. As described above, tissue specificity of ILC2s is acquired through signals from the tissue niches where they reside.

### Localization of ILC2s

Although ILC2s are present in various peripheral tissues, ILC2s are enriched in mucosal organs, such as the skin, intestines, and lungs. In these tissues, ILC2s localize right below epithelial cells and are activated by IL-33, which is released from damaged epithelial cells due to parasites or allergens and induces eosinophilic inflammation. In addition, IL-25 produced by tuft cells activates ILC2s and induces enhanced mucus production [2, 28, 29]. Three-dimensional imaging technologies have shown that ILC2s reside in perivascular adventitial cuff spaces, which represent the outermost layer of blood vessels, in multiple sights including the lungs, liver, brain meninges, and adipose tissue. ILC2s are localized with dendritic cells, regulatory T cells, and adventitial stromal cells (ASCs), a mesenchymal fibroblast-like subset expressing IL-33 and thymic stromal lymphopoietin (TSLP) [30]. In addition, pulmonary neuroendocrine cells (PNECs) reside close to ILC2s near airway branch points in the lungs and amplify allergic asthma responses by producing calcitonin gene–related peptide (CGRP) [31]. Single-cell RNA sequencing studies have shown that intestinal ILC2s are regulated by neuropeptide alpha-calcitonin gene–related peptide (α-CGRP) [32]. As described above, tissue-resident ILC2s localize with epithelial cells, immune cells, neurons, and mesenchymal cells in peripheral tissues. We will explain the interaction between ILC2s and these cells below.

### Epithelial cells and ILC2s

**Fig. 2:**
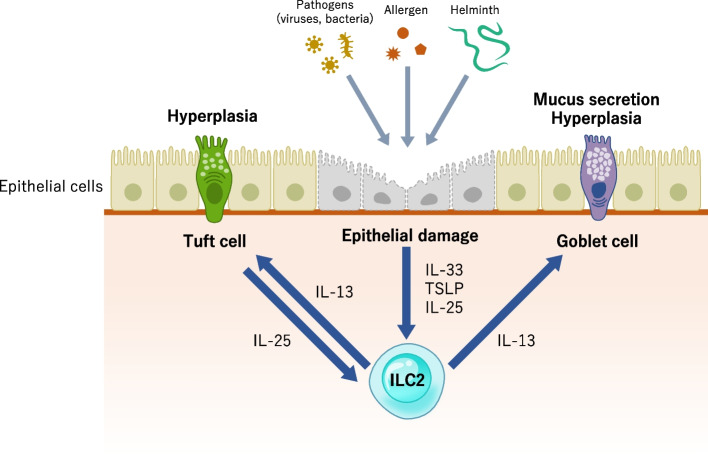
Regulation of ILC2s by epithelial cells. ILC2s reside just below the epithelial cells in mucosal tissues, such as lung, intestine and skin. IL-33, IL-25, and TSLP are released from epithelial cell in response to epithelial damage due to invasion of allergen, helminth and pathogen. These alarmins activate ILC2s and lead to type 2 inflammation

Epithelial cells in the skin and mucosal tissues, including the lungs and intestines, constitute a barrier between the external environment and the underlying mesenchyme. They also respond to external stimuli and interact with immune cells to maintain homeostasis (Fig. 2).

The epithelial cell–derived danger signal mediators IL-33, TSLP, and IL-25 are consistently associated with type 2 immune responses in allergic diseases [33]. Genome-wide association studies (GWAS) have shown an association between allergic diseases and genetic polymorphisms in genes such as *TSLP*,* IL33*, and *IL1RL1,* which encode the IL-33 receptor ST2 [34]. IL-33 is normally found in the nucleus of epithelial cells. When activated or damaged by exposure to parasites or allergen-derived proteases, full-length IL-33 is released from epithelial cells and rapidly processed into a mature active form, which leads to strong ILC2 activation [35]. Although studies have reported that structural cells, including endothelial cells, fibroblasts, smooth muscle cells, and epithelial cells, express IL-33 in various organs, bronchial epithelium is reported to be an important reservoir of IL-33 in the lungs and expression levels are elevated in patients with bronchial asthma [36]. TSLP expression is also higher in the airways of patients with asthma than in those of healthy controls. TSLP levels correlate with Th2 cytokine and chemokine expression and disease severity [37, 38]. TSLP alone mildly activates ILC2s; however, when administered with IL-33, TSLP induces ILC2 corticosteroid resistance by controlling phosphorylation of STAT5 [39]. Because corticosteroid resistance contributes to uncontrolled severe asthma and TSLP upregulates antigen-specific Th2 cell cytokine production through its activity on innate immune cells as well as dendritic cells, T cells, and B cells, a human monoclonal antibody specific for TSLP has been developed [40]. IL-25 is also released from epithelial cells. Allergen provocation induces increased expression of IL-25 and its receptor in the bronchial mucosa and dermis of patients with asthma or atopic dermatitis [41]. In addition, tuft cells, a cell type in the epithelium of the small intestines, express IL-25 to sustain ILC2 homeostasis in a steady state. During helminth infection, IL-25 derived from tuft cells induces IL-13 secretion from ILC2s, which leads to epithelial remodeling by increasing the number of tuft and goblet cells [29]. Moreover, E-cadherin, an adhesion protein expressed by epithelial cells that is responsible for maintaining the integrity of the epithelium, acts as a ligand for KLRG1 and suppresses ILC2 function to avoid excessive ILC2 activation [17]. In inflamed skin lesions of patients with atopic dermatitis, cleavage of E-cadherin might lead to the discontinuation of the inhibitory signal from ILC2s.

### Neurons and ILC2s

**Fig. 3:**
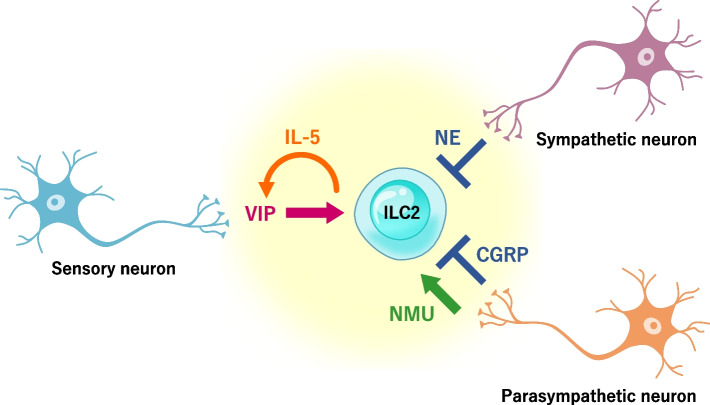
Regulation of ILC2s by nervous system. Sensory, sympathetic and parasympathetic neurons regulate the function of ILC2 by various neurotransmitters. Inhaled allergen or food intake stimulates sensory neurons to produce VIP in the lung and intestine respectively. VIP induces IL-5 production by ILC2s, which in turn enhances VIP production. During helminth infection, NE from sympathetic neurons inhibit cell proliferations and cytokine production in both intestine and lung ILC2s. NMU from parasympathetic neuron enhances the proliferation and cytokine secretion by ILC2s, while CGRP suppress the proliferation and IL-13 production from ILC2s

The immune and nervous systems can communicate using common molecular signaling cues in various organ systems. Recent studies have revealed that ILC2s express receptors for several neurotransmitters, such as vasoactive intestinal polypeptide (VIP), neuromedin U (NMU), CGRP, and norepinephrine (NE). ILC2s are directly activated and regulated by these neurotransmitters produced by various peripheral neurons (Fig. 3).

The peripheral nervous system is composed of the somatic nervous system, which includes motor and sensory neurons, and the autonomic nervous system, which includes sympathetic and parasympathetic neurons. Sensory, sympathetic, and parasympathetic neurons are reported to regulate ILC2s independently from cytokine stimuli.

Sensory neurons transmit sensory information about the body and internal organs to the central nervous system. The lungs have a dense network of nociceptors expressing sensory neurons, which produce VIP and exacerbate asthma symptoms by activating T cells and ILC2s via the VIP–VIP receptor type 2 axis. IL-5 produced by activated ILC2s enhances VIP production, creating a positive feedback loop between sensory neurons and ILC2s [42]. Sensory neurons in the small intestines also produce VIP when stimulated by nutrient intake and central circadian rhythms, leading to the induction of IL-5 production by ILC2s and contributing to basal eosinophilopoiesis and eosinophil accumulation in tissues [43].

Sympathetic neurons control the fight-or-flight response in stressed, dangerous, or physically active situations. They increase heart rate and promote vasoconstriction to activate physical activity while suppressing intestinal motility and intestinal tract secretion. The sympathetic nervous system includes adrenergic neurons that produce catecholamines, i.e., epinephrine and NE. Catecholamines exert their effect via two classes of adrenergic receptors α (α1, α2) and β (β1, β2, and β3). ILC2s express the β2-adrenergic receptor (β2AR) and colocalize with adrenergic neurons in the intestines. Moriyama et al. reported that the β2AR pathway is a cell-intrinsic negative regulator of ILC2 responses via inhibition of cell proliferation and effector function [44].

Parasympathetic neurons are responsible for stimulation of rest-and-digest or feed-and-breed activities when the body is at rest. They play an antagonistic role to sympathetic neurons. They reduce heart rate, relax blood vessels, and activate digestive activity. The parasympathetic nervous system mainly uses acetylcholine as its neurotransmitter, which is produced by cholinergic neurons. NMU is produced by cholinergic neurons and signals through its receptors neuromedin U receptor 1 (NMUR1) and NMUR2 [45]. Single-cell RNA sequencing and genome-wide transcriptional profiling have revealed that ILC2s selectively express *Nmur1*. ILC2s in the murine gastrointestinal tract colocalize with cholinergic neurons that express NMU. NMU–NMU1 signaling induces cell activation, proliferation, and secretion of the type 2 cytokines IL-5, IL-9, and IL-13 from ILC2s. Worm products and alarmins directly stimulates mucosal neurons to induce NMU and lead to accelerated expulsion of *Nippostrongylus brasiliensis*. Lung ILC2s express NUMR1 in the steady state and after IL-25 stimulation; in vivo co-administration of NMU with IL-25 strongly amplifies allergic inflammation [46–48]. Of note, NMU induces both smooth muscle contraction [49], which exacerbates asthma, and ILC2-driven inflammation. On the other hand, CGRP released from parasympathetic neurons suppresses the proliferation of ILC2s and IL-13 production by ILC2s and attenuates type 2 inflammation in the lungs and intestines [32, 50, 51].

In summary, the nervous system appears to have two mechanisms to rapidly activate or repress these innate immune cells in order to maintain tissue homeostasis and protect the host against diverse inflammatory stimuli. These findings highlight the importance of neuro–immune crosstalk at mucosal surfaces. The investigation of neuroimmune interactions with ILC2s might lead to greater understanding of the mechanisms of asthma exacerbation by non-antigenic factors such as air pollutants, cold exposure, and psychological stress.

### Immune cells and ILC2s

**Fig. 4:**
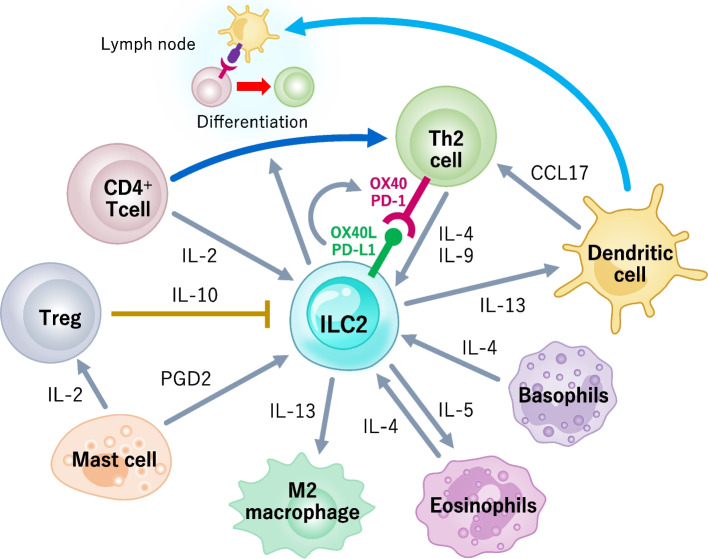
Regulation of ILC2s by immune cells. ILC2s interact with various immune cells by cytokines, lipid mediators, and cell–cell contact. ILC2s and Th2 cells compound positive feedback loop driving innate and adaptive immune responses in allergic inflammation and anti-helminth immunity. In visceral adipose tissue, IL-5 and IL-13 produced from ILC2s promote eosinophils and M2 macrophages implicated in metabolic homeostasis

ILC2s interact with various immune cells via cell–cell contact or communication via soluble factors such as cytokines, lipid mediators, and hormones (Fig. 4). ILC2s colocalize with regulatory T (T_reg_) cells, CD4^+^ T helper type 2 (Th2) cells, and dendritic cells (DCs) in the perivascular adventitial cuff of peripheral tissues.

ILC2s drive the initiation of type 2 innate immune responses, leading to the activation of the adaptive immune system driven by Th2 cells. CD4^+^T cells provide IL-2, which promotes ILC2 proliferation and IL-13 production. ILC2s, in turn, influence the differentiation of naïve CD4^+^ T cells into a Th2 phenotype in a contact-dependent manner [52]. Moreover, activated ILC2s upregulate the expression of OX40 ligand and programmed death ligand 1, leading to the activation of Th2 cells by upregulating GATA3 expression and cytokine production [53, 54]. Activated Th2 cells produce IL-4 and IL-9, which contribute to ILC2 activation. This positive feedback loop drives both innate and adaptive immune responses. In addition to Th2 cells, basophil- and eosinophil-derived IL-4 enhances ILC2-derived cytokine and chemokine expression, leading to type 2 inflammation [55, 56]. IL-5 produced by ILC2s enhances the proliferation, survival, and recruitment of eosinophils [43]. Eosinophils, in turn, produce IL-4 to stimulate ILC2s, thereby mediating the crosstalk between eosinophils and ILC2s.

*T*_reg_ cells have the capacity to mediate suppression of a variety of immune cells and exert anti-inflammatory effects. In addition to the production of suppressive cytokines TGF-β and IL-10, *T*_reg_ cells suppress ILC2s by inducible T cell costimulator (ICOS)–ICOS ligand interaction [57]. IL-33 induces mast cells to secrete a variety of inflammatory mediators, including IL-2. IL-2 secreted by mast cells promotes an increase in the number of IL-10–producing *T*_reg_ cells and suppresses IL-33–induced airway eosinophilia, constituting an anti-inflammatory negative feedback system [58]. On the other hand, mast cell–derived prostaglandin D2 activates ILC2s via its receptor, chemoattractant receptor–homologous molecule expressed on TH2 cells (CRTH2), and mediates strong proallergic inflammatory responses [59].

DCs are a type of antigen-presenting cell. They play an essential role in promoting the adaptive immune response. ILC2-derived IL-13 promotes DC migration into draining lymph nodes, where DCs prime naïve T cells to differentiate into Th2 cells [60]. ILC2-derived IL-13 also induces the production of CCL17 by DCs, which promotes the recruitment of CCR4^+^ memory Th2 cells [61].

In addition, ILC2s interact with macrophages. In the context of polarized type 2 immune responses, macrophages assume a distinct state of alternative activation into M2 macrophages, which have critical functions in allergic inflammation, helminth infection, and maintenance of metabolic homeostasis. Type 2 cytokines, especially IL-13, produced by activated ILC2s promote M2 polarization, resulting in protective immunity in a cerebral malaria model and helminth infection, or induce allergic inflammation in fungal infection [62–64]. Moreover, M2 macrophages in visceral adipose tissue (VAT) play an important role in glucose and fat metabolism. VAT-resident ILC2s are the major source of IL-5 and IL-13; they promote the accumulation of eosinophils and M2 macrophages. These cells are required for protection from increased adiposity and insulin resistance in the context of a high-fat diet [65].

### Mesenchymal cells and ILC2s

**Fig. 5:**
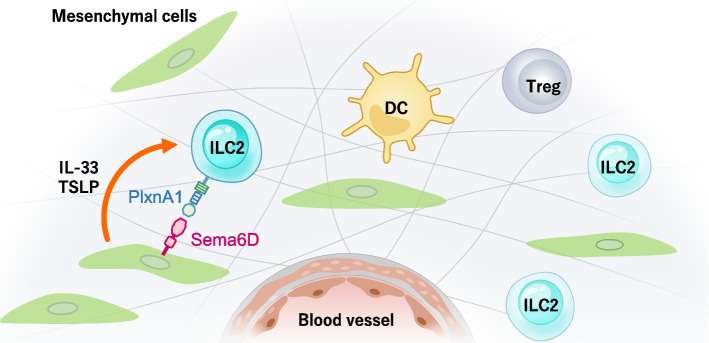
Regulation of ILC2s by mesenchymal cells. ILC2s localize with fibroblast-like mesenchymal cells in perivascular regions. Mesenchymal cells produce IL-33 and TSLP to support ILC2s, and ILC2s promote mesenchymal cell expansion and IL-33 production during helminth infection. Mesenchymal cells not only activate ILC2s but also regulate IL-10 production via Semaphorine6D-PlexinA1 manner

ASCs, a platelet derived growth factor receptor alpha (PDGFRα) low expressing fibroblast-like stromal cells, produces IL-33 and TSLP to support ILC2s in the peripheral microenvironment in various tissues (Fig. 5). ILC2s, in turn, promote ASC expansion and IL-33 production after helminth infection. Single-cell RNA sequencing has revealed that ASCs express high levels of collagens and cytokines and that they are associated with extracellular matrix remodeling and immune responses [30]. In adipose tissue, white adipose tissue–resident multipotent stromal cells (WAT-MSCs) act as a reservoir of IL-33 to sustain ILC2s. WAT-MSCs also support the proliferation and activation of ILC2s in an ICAM-1–LFA-1 manner.

A recent study revealed that lung ASCs regulate IL-10 production in ILC2s via the semaphorin 6D (Sema6D)–plexin A1 axis [66]. Semaphorins, which are axon guidance molecules, were originally identified during neuronal development. They have various effects on angiogenesis, tumor growth, bone homeostasis, and immune responses [67–71]. Sema6D-expressing ASCs suppress IL-10 production by ILC2s in vitro. Deletion of Sema6D ameliorates acute lung inflammation caused by IL-33 and *Alternaria alternata*.

## Conclusions

Progress in the past several years has clarified that ILC2s are highly dynamic cells that can migrate from tissue to tissue and adapt their effector functions depending on the environment in which they reside. As mentioned earlier, in contrast to T cells and B cells, ILC2s lack antigen-specific receptors. Instead, ILC2s are directly regulated by various mediators including cytokines, lipid mediators, neurotransmitters, and cell–cell contact. Moreover, as ILC2s are tissue-resident cells, such mediators in the local microenvironment have a significant effect on the function, localization, and phenotype of ILC2s. In this review, we summarized the current knowledge regarding the mechanisms that regulate ILC2s from the perspective of the cells in the surrounding environment.

Understanding of how ILC2s are regulated highlights the therapeutic importance of targeting the surrounding mediators. For example, along with Th2 cells, ILC2s are a well-known component of an immune cell network that contributes to the pathological state of asthma. Biologics targeting IgE, IL-4, IL-5, IL-13, and TSLP are used for uncontrolled severe asthma; their targets are all mediators produced by cells in the microenvironment of ILC2s. Blood eosinophil count, fractional exhaled nitric oxide (FeNO), and blood IgE titer are used as diagnostic biomarkers for severe asthma [72–74]. Among these biomarkers, blood eosinophil count and FeNO are reported to be useful for predicting the efficacy of biologics in patients with severe asthma [75–78]. ILC2s produce IL-5 and enhance the proliferation, survival, and recruitment of eosinophils, which can be monitored by blood eosinophil count. However, human blood ILC2 count it is usually not measured clinically. A basic science study has shown that patients with asthma have more blood ILC2s which produce more IL-5 and IL-13 than healthy controls [79], indicating that the evaluation of blood ILC2 in patients with asthma might be clinically useful. Clinical application of ILC2 measurement in humans is a task for the future. In addition to surrounding mediators of ILC2s, regulatory functions of ILC2 might be the key to treating allergic diseases. It has been reported that allergen-specific immunotherapy restores the ability of ILC2s to produce IL-10, and IL-10–producing ILC2s play a critical role in the induction of tolerance to aeroallergens [80]. Mesenchymal cells, which regulate IL-10 production by ILC2s, might be a potential treatment target in allergic diseases.

The location of ILC2s differs between humans and mice. Moreover, ILC2s have different cytokine production profiles, receptor expression, and signaling pathways among species. Although mouse studies and human studies complement each other, we have to carefully interpret these differences.

ILC2s have been identified to be involved in many diseases related to excessive activation or dysfunction of tissue-repairing and maintaining tissue-homeostatic balance. Further clarification of the mechanisms by which ILC2s regulate the immune system will certainly shed light on the development of novel therapeutic approaches for these diseases.

## Data Availability

Not applicable.

## Abbreviations

ASC: Adventitial stromal cell
CGRP: Calcitonin gene–related peptide
CRTH2: Chemoattractant receptor–homologous molecule expressed on Th2 cells
DC: Dendritic cell
FeNO: Fractional exhaled nitric oxide
GATA3: GATA-binding protein 3
GWAS: Genome-wide association studies
ICOS: Inducible T cell costimulator
ILC2: Group 2 innate lymphoid cell
NE: Norepinephrine
NMU: Neuromedin U
NMUR1: NMU receptor 1
NMUR2: NMU receptor 2
Nrp1: Neuropilin-1
PNEC: Pulmonary neuroendocrine cell
Sema6D: Semaphorin 6D
S1p: Sphingosine-1-phosphate
TGFβ1: Transforming growth factor beta-1
Th2: CD4^+^ T helper type2 cell
T_reg_: Regulatory T cell
TSLP: Thymic stromal lymphopoietin
VAT: Visceral adipose tissue
VIP: Vasoactive intestinal polypeptide
WAT-MSC: White adipose tissue–resident multipotent stromal cell
α-CGRP: Alpha-calcitonin gene–related peptide
β2AR: β2-Adrenergic receptor
